# Genetic and Survey Data Improves Performance of Machine Learning Model for Long COVID

**DOI:** 10.21203/rs.3.rs-3749510/v1

**Published:** 2023-12-19

**Authors:** Wei-Qi Wei, Christopher Guardo, Srushti Gandireddy, Chao Yan, Henry Ong, Vern Kerchberger, Alyson Dickson, Emily Pfaff, Hiral Master, Melissa Basford, Nguyen Tran, Salvatore Mancuso, Toufeeq Syed, Zhongming Zhao, QiPing Feng, Melissa Haendel, Christopher Lunt, Geoffrey Ginsburg, Christopher Chute, Joshua Denny, Dan Roden

**Affiliations:** Vanderbilt University Medical Center; Vanderbilt University Medical Center; Vanderbilt University Medical Center; Vanderbilt University Medical Center; Vanderbilt University Medical Center; Vanderbilt University Medical Center; Vanderbilt University Medical Center; University of North Carolina, USA; Vanderbilt University Medical Center; Vanderbilt Institute of Clinical and Translational Research/Vanderbilt University Medical Center; Stanford University School of Medicine; Stanford University School of Medicine; UTHealth Houston; University of Texas HSC Houston; Department of Medicine, Vanderbilt University Medical Center; University of Colorado; All of Us Research Program; All of Us Research Program, National Institutes of Health; Johns Hopkins University; All of Us Research Program, National Institutes of Health; Department of Biomedical Informatics, Vanderbilt University Medical Center, Nashville, TN

## Abstract

Over 200 million SARS-CoV-2 patients have or will develop persistent symptoms (long COVID). Given this pressing research priority, the National COVID Cohort Collaborative (N3C) developed a machine learning model using only electronic health record data to identify potential patients with long COVID. We hypothesized that additional data from health surveys, mobile devices, and genotypes could improve prediction ability. In a cohort of SARS-CoV-2 infected individuals (n=17,755) in the All of Us program, we applied and expanded upon the N3C long COVID prediction model, testing machine learning infrastructures, assessing model performance, and identifying factors that contributed most to the prediction models. For the survey/mobile device information and genetic data, extreme gradient boosting and a convolutional neural network delivered the best performance for predicting long COVID, respectively. Combined survey, genetic, and mobile data increased specificity and the Area Under Curve the Receiver Operating Characteristic score versus the original N3C model.

## INTRODUCTION

Post-acute sequelae of SARS-CoV-2 infection (i.e., long COVID) is a growing public health crisis globally.^1^ There have been over 769 million confirmed COVID-19 cases worldwide, and more than 200 million individuals infected have had or will develop persistent symptoms of COVID-19.^1,2^ Previous studies have highlighted the presence of a broad spectrum of clinical, social, and psychological consequences following primary infection with the SARS-CoV-2 virus.^3^ Despite the public health importance, long COVID remains largely unexplained and understudied.

Given the complexity and variety of its presentations, one of the persistent challenges hindering long COVID research has been the successful identification of cases. As such, the National COVID Cohort Collaborative (N3C) collaboratively collected a large electronic health record repository of SARS-CoV-2-infected individuals. The group developed an advanced machine learning (ML) based phenotype for long COVID and tested it using the NIH’s All of Us (AoU) data.^3,4^ While an important step in identifying long COVID patients, the model relies entirely on patients’ EHR data, which is only representative of a patient’s recorded history in a medical setting. This potentially limits the algorithm’s ability to detect cases.

COVID research is rapidly evolving; indeed, since the original N3C model was published in 2022, researchers have identified several factors that are indicative of the presence of long COVID. Multiple studies have shown that long COVID has a significant association to a person’s genetic. For example, a recent large genome-wide association analysis demonstrated that the Forkhead Box P4 gene (*FOXP4*) has a significant association to long COVID.^5^ Further, there have been over 20 genetic variants identified with significant associations to COVID-19 contraction and hospitalization.^6^ Given these findings, it is plausible that these genetic variants may also have an effect on a person’s risk to have long lasting side effects after contracting COVID-19.

Along with genetic factors, researchers have also identified that social and lifestyle factors captured in survey and mobile device data may be associated with COVID-19 (and thus could be used to improve models). For example, findings among AoU research participants showed a statistically significant decrease in step count before and after the COVID-19 pandemic and vulnerable populations, including individuals at a lower socioeconomic status were at the highest risk of reduced activity.^7^ Further, studies demonstrated that external factors (e.g., socioeconomic indicators) are associated with the diagnosis and treatment of long COVID.^8^ Because social determinants of health (SDOH) contribute to a persons’ susceptibility to long COVID, our team aims to leverage survey and mobile device data available in the AoU platform, which include features from four of the five domains of social determinates of health. ^9^

Given the plausibility of genetic, social, and lifestyle factors’ association with long COVID, we hypothesized that the integration of genotypes, mobile device information, and health survey data into a predictive model could improve the performance of the existing EHR-based algorithm. One of the challenges facing algorithm development has been the perceived requirement of extensive data sharing or repeatedly developing algorithms with different datasets. We sought to expedite this process by utilizing and further developing an existing algorithm. Our objective was to validate and further enhance the predictability of long COVID by leveraging the genetic, mobile device, and survey data available through AoU Workbench by building from the existing machine learning infrastructure provided from the N3C algorithm.

## RESULTS

### Study population

Our final cohort included 17,755 AoU participants out of which 976 had long COVID. The data availability for the cohort is represented in Fig. 3. Our genetic model was trained on a total of 3,145 participants with 55 long COVID cases. The survey and mobile device model was trained with a total of 14,368 participants, 907 of which were long COVID cases. The gold standard test cohort included 944 participants who had both genetic and survey-based data. Of these participants, < 20 participants were long COVID cases.

### Machine Learning Infrastructure Selection and Combined Model Weighting

Our findings showed that XGBoost was the most effective method for representing survey-based data, and CNN was the most effective method for modeling genetic data to predict a long COVID diagnosis.

When we calculated optimal weights for the combined model parameters, the function found the combination of weights giving the best AUROC score for the preliminary testing set:

[α,β,γ]=[0.58,0.05,0.37]

### Model Results

We used the 100 predictions generated in cross validation to create average AUROC Curves (Fig. 4). We also assessed average positive predictive value, specificity, and sensitivity for each of the models (Table 1).

The AUROC average improved when new data type was added to the algorithm’s predictive method. The addition of survey and mobile device data to the prediction produced the largest increase, raising the AUROC score from an average of 0.721 to an average of 0.842. This increase was significant (p = 0.01) in a two-sample t-test. The combined addition of survey, mobile, and genetic data to the prediction further raised the average AUROC score to 0.855, but this increase was not found to be a statistically significant difference.

While Sensitivity was held as a constant, the specificity also increased for predictive methods that used more data sources. The N3C originally had an average specificity of 0.700. Our final model using EHR, genetic, survey, and mobile device data has a corresponding specificity of 0.867. From this, we see that the predictive method grew in specificity with the addition of new data sources, and that our final predictive model grew drastically in its ability to specify between long COVID cases and controls.

### Fairness Analysis

Access to healthcare and social determinates can often contribute not only to a person’s likelihood to receive a diagnosis, but further their likelihood to be accurately categorized by a machine learning based predictive algorithm. For this reason, we tested our machine learning model’s performance across a few social determinants that were available in the AoU survey questionnaire to see if the model performed better on certain cohorts of participants. We tested performance of our model across sexual orientation, annual income, and education level.

We found significant differences in the model’s performance in participants whose annual income is greater than 50,000 US dollars (average AUROC = 0.841) and participants whose annual income is less than or equal to 50,000 US dollars (average AUROC = 0.947). Similarly, we found that our model performed better on participants who have not attended college for at least one year of their life (average AUROC = 0.908) than participants who have attended a full year of college (average AUROC = 0.840). We found a small but not significant change in model performance between straight participants (average AUROC = 0.845), and sexual minority participants (average AUROC = 0.925).

### Features

Along with assessing models with additional information, we also identified the twenty features that contributed the most predictive capacity to the models (Fig. 5). Of the twenty features with the most relative proportionate contribution to the prediction, ten of the features were existing features from the N3C XGBoost model using EHR data.^3^ These features also comprised all of the top three features in relative proportionate contribution which were post covid outpatient utilization, age, and post covid inpatient utilization. The other ten features with high feature importance all came from the survey and mobile device data model. The survey and mobile device features with the strongest positive correlation of this set were how many years a participant has been living in their current home, how many people under the age of 18 live with the participant, and the participant’s frequency of alcohol consumption in the last year. Features with a negative correlation included Average Pain in the last 7 days, median steps after a positive COVID19 diagnosis, general health rating, general social health rating, and average heart rate after a positive COVID19 diagnosis.

## DISCUSSION

Our findings show that combining multiple data sources using a weighted averaging method can increase the overall accuracy of the existing predictive N3C predictive algorithm. The inclusion of different data types in AoU led to a direct increase in the AUC ROC score of our model. The approach (i.e., using an existing model, but adding new data sources by creating independently run models) allowed us to incorporate the existing predictive strength of the N3C model, which had a larger and more robust training group for EHR data. Additionally, the functional interpretation of our algorithm’s features shows that predictive models incorporating characteristics captured in AoU survey/mobile data improve performance most. When isolating the factors contributing most to the combined predictive model, the N3C components remained the most significant, but the survey and mobile data also played a substantive role. Notably, the genetic variants were not among the top contributors.

The inclusion of different data types led to improved accuracy of our overall prediction, even with data types being trained and modeled separately. These results show that well-crafted existing models can be improved by adding predictors from a smaller, more selective dataset in certain circumstances. The success of this method (i.e., starting with an original model created using a powerful dataset, and adding new independently trained models incorporating more recently available new datatypes) demonstrates that we can leverage existing Machine Learning algorithms and improve on the predictive outcome using new data types without requiring extensive data sharing or repeated algorithmic development.

The models exhibited fairness for predicting long COVID risk across various metrics by sexual orientation, annual income, and education level. For instance, both the AUROC and PPV were both higher among sexual minority participants compared to straight participants and the overall sample. This may be attributed to the deliberate oversampling of individuals from underrepresented communities in biomedical research by AoU, highlighting the significance of representation from marginalized communities in developing prediction algorithms to enhance model fairness. However, it is important to recognize that evaluating fairness based on binary demographic characteristics is an oversimplification. Health inequities often result from complex systems of intersecting power (e.g., heterosexism, racism) and necessitate an intersectional approach to evaluating model fairness when deploying algorithms to ensure equitable outcomes in mitigating the risk of long COVID.^10–13^

Many phenotypes have utilized Health Survey Data in AoU to increase the scope of data utilized for predictive models. Health survey data in AoU is collected on enrollment in the study and covers a wide range of information including overall health, living situation, education, income, alcohol and drug usage, home ownership, marital status, sex, insurance, and birthplace.

Although long COVID has considerable health concerns for many patients, there are striking differences between the diagnosis and treatment of long COVID.^14^ Due to this challenge, an application of a machine learning algorithm could be a valuable clinical aid to identifying patients with long COVID and assessing susceptibility among a wide range of patients. Further, the interpretation of feature contributions in our predictive model highlights the relative high importance of survey data and a lesser observable impact of known genetic factors to a person’s risk for long COVID. By implementing similar surveys in hospitals, our algorithm could be used as a screening tool for clinicians to anticipate long term health complications for patients at risk for long COVID.

This study has some limitations. Our original models were trained in part to make use of the output from the N3C teams machine learning model. Because of this, the biases and limitations present in the N3C model likely carry over to our predictive method. Our algorithm based on AoU data has not been validated with any external datasets. Further there are many limitations on the data used to create our algorithm. We only used preidentified COVID-19 related genetic variants instead of whole genome sequencing data. As such, the genetic contribution is limited known genetic indicators of COVID-19 hospitalization and contraction. This validation of this predictive method represents a particular challenge given that the survey data required for assessment limits the overall study cohort; however, these findings may also encourage the collection of this type of data on a broader scale. AoU data contains its own limitations as well, including an age restriction of > = 18 years, non-universal observational periods for each participant, and occasional missing data points (more prominent in the survey data). Finally, because long COVID is a relatively new disease, diagnosis in a patient’s medical history is uncertain, potentially causing variability in the accuracy of our data.

Despite these limitations, this algorithm can improve the power of predicting long COVID risk for patients by leveraging genetic, survey, mobile device, and EHR data. Further, the method for our algorithm’s development reached some success in improving the performance of an existing model using new data. This means others could use the same method to improve the performance of an existing model by leveraging the power of new data sources as they become available.

## METHODS

### Data Source and Cohort

Data for this study were derived from the AoU data enclave (version 7) available in the preproduction environment,^9^ which consist of EHRs, survey-based information, genotyping data, and lifestyle data (collected through mobile devices) of non-deceased adults (18 years or older) living in the United States. EHR data encompass demographics, health-care visit details, medical conditions, and prescription drug orders for each patient.

Our cohort inclusion criteria largely paralleled that used for the original N3C study.^3^ Specifically, cohort members were required to have either 1) an International Classification of Diseases, Tenth Revision, Clinical Modification (ICD-10-CM) COVID-19 diagnosis code (i.e., U07.1) from an inpatient or emergency health-care visit, or 2) a positive SARS-CoV-2 PCR or antigen test.

We required patients to have EHR data available in AoU before and after the diagnosis date or positive test result. We excluded patients with < 90 days between the positive COVID-19 test or diagnosis date and the end of the study (May 2023) to ensure sufficient follow-up to assess long COVID.^3^ To accommodate the existing EHR data model, we gathered EHR data from AoU participants. We only gathered variables that were used in the pre-existing models and made only the exact same transformations given from the previous literature’s data cleaning protocol.^3^ We used EHR data in the AoU preproduction environment, which follows the common OMOP standard data model. The only additional inclusion criterion was that patients must have valid data (described below) for least one other data type (i.e., genetic, survey, or mobile device data).

From the genetic data, we used discrete variables showing the minor allele counts (0, 1, and 2) for each genetic variant. We extracted 25 genetic variants from AoU whole genome sequencing (WGS) data version 7 (**Supplemental Table 1**). These variants were effect alleles identified by the COVID-19 Genetic Consortium and were significantly associated to COVID-19 infection or hospitalization due to COVID-19.^6^ Participants with genetic data were included only if they had non-null values for the genetic expression of each of our pre-specified 25 genetic variants (**Supplemental Table 1**).

From the health survey information available in AoU, we selected 32 questions which gave information that was distinctively different from the data available through our EHR data (**Supplemental Table 2**). These questionnaire outputs were transformed into scalar variables for model testing and training. Participants considered to have valid survey data must have completed the AoU core survey questionnaire (the basics, lifestyle, and overall health) within the time span of their EHR history in AoU. Further, participants who skipped 10% or more of the questions used for our algorithms were excluded.

The mobile device data collected in AoU derives from patients’ Fitbits or other smart devices, which provide measures such as heart rate, steps, and sleeping status. Several factors contributed to variability in data availability. First, patients varied in the consistency of their monitoring. Second, the timing of the COVID-19 diagnosis relative to the span of available data differed. For instance, if a patient received a COVID-19 diagnosis 3 months into a year of data, most of the that participant’s data would be after their COVID-19 diagnosis, while others who received a COVID-19 diagnosis later would have proportionally more data before their diagnosis.

Given this variability, our goal was to select a set of features which gave interpretative value to the mobile device data for our models and worked regardless of observation period. We narrowed our inclusion criteria down to only patients with a positive COVID-19 test or diagnosis result available in their EHR history. Long COVID is normally defined as persisting symptoms more than 28 days after a positive COVID-19 test, and tests are relatively accurate up to a week of COVID-19 contraction. Because of this, we excluded all mobile device measurements between 7 days before the COVID-19 test to 28 days after the COVID-19 test. We then created averages for the total measurements before and after this excluded time frame. For example, step counts from a patient’s mobile device data were extracted into two features for our machine learning model. The first feature measures the patients average step count per day from the start of the survey to 7 days prior to their first COVID-19 positive test. The second feature measures the patient’s average step count per day starting 28 days after the positive COVID-19 test (**Supplemental Table 3**).

Participants with mobile device data were considered only if they had sufficient data points both before and after a COVID-19 test or diagnosis date in their EHR. We required patients to have two or more data points earlier than seven days prior to their COVID-19 test or diagnosis, and two or more data points following 28 days after their positive COVID-19 test or diagnosis date. Further, participants’ measures had to fall within a clinically reasonable average range for heart rate, step count, and average minutes asleep for us to consider their mobile device data reasonable. Specifically, we applied exclusion criteria based on the reasonability of a participants’ average health metrics, by giving each variable a range and excluding data that fell over that range. The reasonable ranges for each metric are as follows: average heart rate < = 100bmp, minimum heart rate (for a single day) < = 125bpm, median minutes asleep < = 600, and median steps < = 100000.

Data cleaning, feature engineering, and model training were completed using AoU Researcher Workbench cloud computing.

### Training, Testing, and Validating Sets

**Figure 1:**
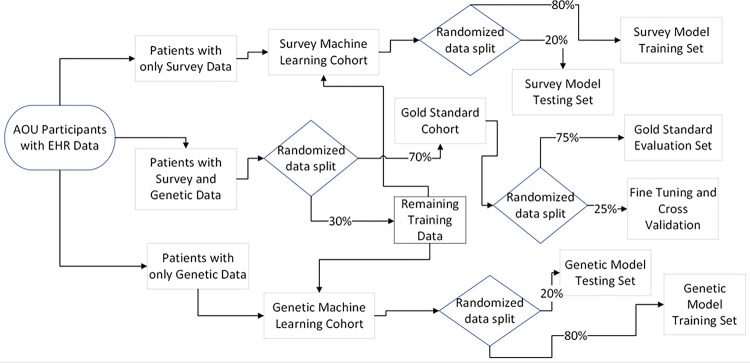
Training, Testing, and Validation Set Parameters. Flowchart describing how the AoU data was divided into sets based on data availability.

We first reserved the patients with all data sources (i.e., EHR, survey and/or mobile device, and genetic data) for validation. We named this cohort the gold standard testing set, and this set was used to evaluate the combined prediction given from all the model’s combined predictive output. From the remaining participants, we separated patients based on available data, and trained each model with 75% of the patients with that data type available. This data set is referenced later as the training data set. The corresponding 25% of remaining patients with that data were designated as a preliminary testing set. The preliminary testing set was used to test the output of a single model iteratively in the model training process (Fig. 1).

### Transformation of Training Data

While long COVID is a common disease, the associated ICD code U09.9 is quite rare and under-utilized in clinical practice, and it was not available before October 2021. Because long COVID is a rare diagnostic billing codes, the rarity of true cases makes training machine learning models difficult. Given the limited scope of our data, there is a restrictively small number of true long COVID cases to train our models. However, the N3C model was created using a more robust dataset. Therefore, to increase the number of cases in our training set, we also counted patients that the N3C model gave a predictive probability value of > 0.9 as a long COVID case. Thus, we defined long COVID cases for our training set as participants with either (1) the U09.9 diagnostic code or (2) a predicted probability for having long COVID of > 0.9 based on the original N3C EHR algorithm.

Although this definition is helpful for training new models on limited data sets, it is still important to maintain some level of accuracy for our total evaluation set. For this reason, the definition for long COVID in our testing sets remained unchanged, and we evaluated the performance of our models only using the U09.9 diagnostic code for long COVID.

### Model Design and Machine Learning Infrastructure Selection

We implemented the N3C model to determine baseline performance for all data sets. We then trained two models independently: (1) genetic data alone, and (2) survey and mobile device data. We grouped mobile device data and survey data due to their high correlation and aptitude for missing elements. Following the N3C approach, we used the existing XGBoost algorithm for EHR data. We then selected the most appropriate ML infrastructure for the remaining data sources among Logistic Regression, Polynomial Regression, Random Forests, Convolutional Neural Networks, K Nearest Neighbors, Support Vector Machines, and Extreme Gradient Boosting Trees (Fig. 1). We first ran preliminary tests to find the most effective ML infrastructure to represent each data type individually. Our preliminary tests consisted of calculating the AUROC score for each model using preliminary testing data and comparing the base performance of different ML infrastructures. Then a different model was created for each ML infrastructure, and the best infrastructure was picked based on the leading AUROC score for each data type. Given the missingness of the survey and mobile device data, extreme gradient boosting (XGBoost) was the best performing model type for this data. The genetic data alone was found to have the highest AUROC score when using a convolutional neural network (CNN) machine learning infrastructure.

### Predictive Method

The individual predictive outcomes from our independent models were combined using a weighted average function to give a single overall outcome for our combined methodology. Our weighted average determined a person’s likelihood of long COVID by taking each of models results and multiplying it by a given proportional weight. The weights were combined in a sum for overall confidence using the equation below:

$$P\left(X_{total\;patient\;data\;}\right)=\alpha \cdot P_{1}\left(X_{EHR}\right)+\beta \cdot P_{2}\left(X_{genetic\;}\right)+\gamma \cdot P_{3}\left(X_{survey\;and\;mobile\;device}\right)$$

X represents the participant’s data, with subsets of the participant’s data represented by a subscript. P represents the probabilistic outcome of a machine learning model with P1,P2, and P3 representing the outcomes of the EHR, genetic, and survey models, respectively. The non-negative constants α,β, and γ are proportional weights given to the predictive outcome of each model. The total output is given as a percentage that represents the overall predictive method described as a probability. For this reason, α,β, and γ are fitted using the equation below.

α+β+γ=1

These proportional weights were used as a fine-tuning parameter for our predictive method. By taking the sum of these products, the result is a single probability percentage for a patient’s risk for long COVID.

To calculate the optimal weights for these parameters, we designed a function to iterate through possible weights for α,β, and γ. The function found the combination of weights that gave the best AUROC score for the preliminary testing set, accurate to the nearest hundredth.

### Cross Validation

**Figure 2:**
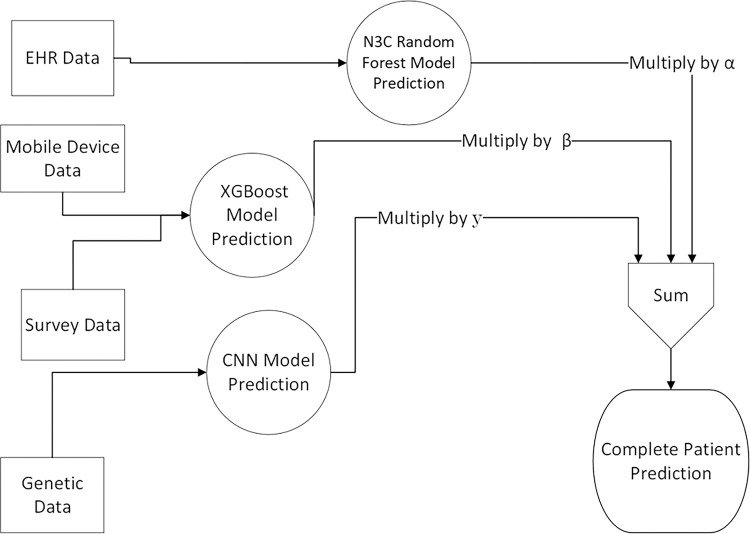
Model Output Sources and Predictive Method. Flowchart showing how the predictive components of separate models are combined into a single predictive outcome.

**Figure 3:**
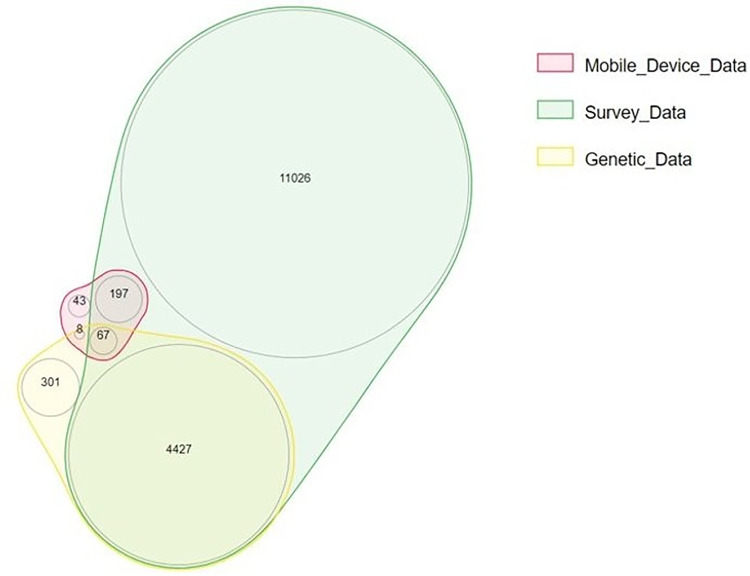
Cohort Data Availability. Venn diagram showing the data available for AoU participants.

**Figure 4:**
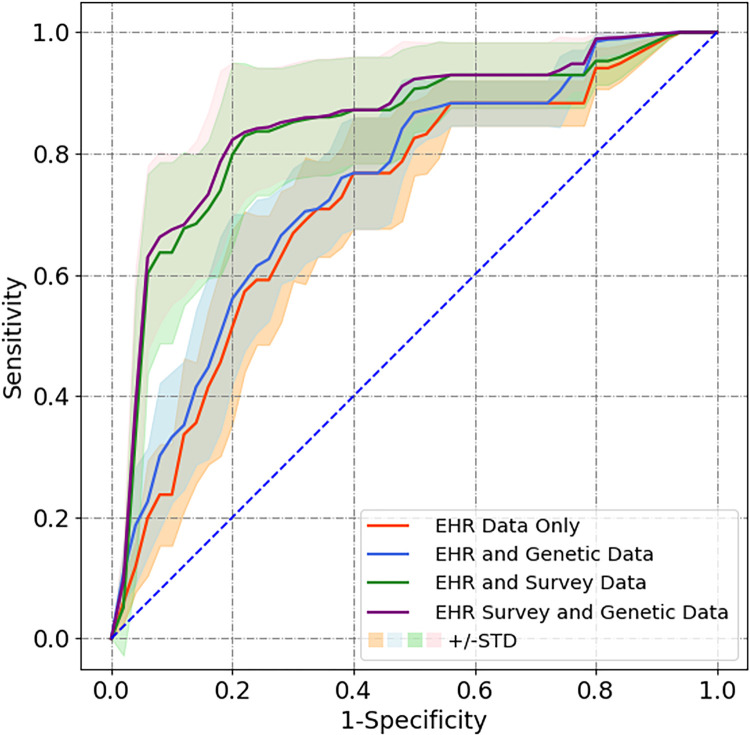
Average ROC of Models. Shows the Average ROC curve for each combination of models from cross validation results. Shaded area represents the standard deviation of the ROC curve, and color represents models used.

**Figure 5:**
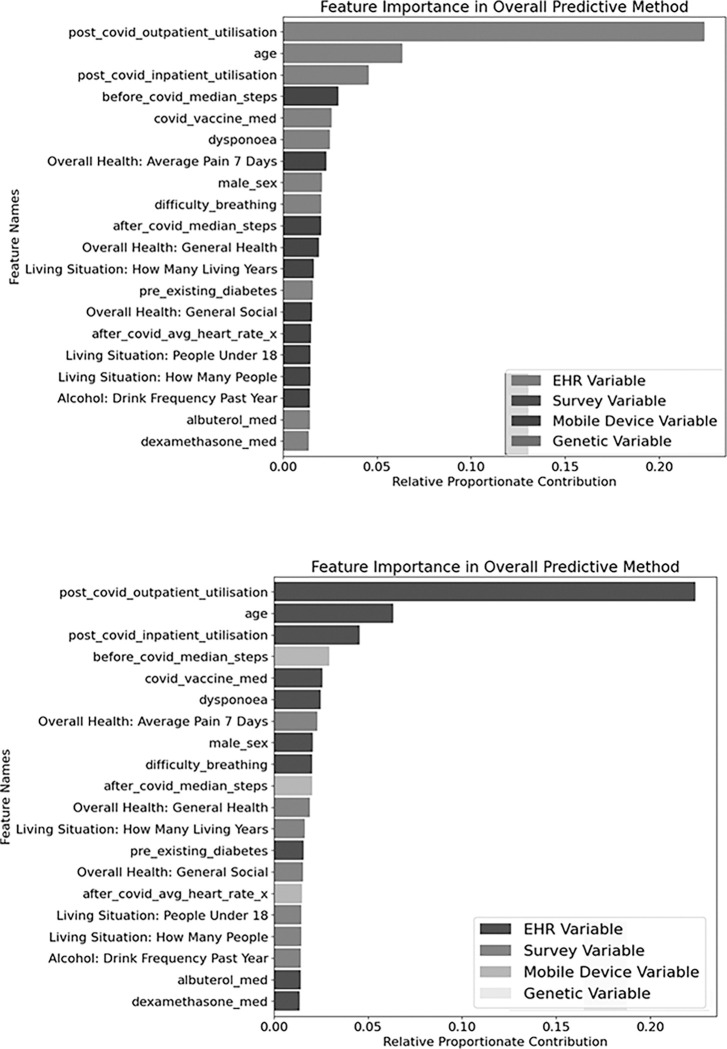
Top Twenty Predictive Features in the Combined Model. The relative contribution of the 20 most powerful features in our predictive method. Color of each bar represents the data source of the feature.

We created a set of parameters for training models and validated our results using an original cross validation method. We trained each model separately using a 5-fold cross validation method. Then, we divided our Gold-Standard Evaluation cohort into 4 groups and cycled through portions of the Gold-Standard Evaluation set to evaluate the prediction given by each combination of models. Because of this, our final validation used 100 unique iterations of our data to train, test, and validate the predictive method (Fig. 2). We assessed AUROC, positive predictive values, specificity, and sensitivity for each iteration and then the average of the 100 iterations for each model. We used a two-sample t-test for difference in sample means to assess the significance of difference between the AUROC.

Further, to assess the difference in the specificity for our predictive models, we created an optimization function to choose a threshold for each model so that the results would have a resulting sensitivity closest 0.7. Then, we did a two-sample t-test for difference in sample means for the specificity of each predictive model’s outcome.

### Assessing Individual Predictive Features

Using Shapely as an analysis tool for ML methods, we determined the most important features in the final predictive method based on Shapley values and the relative direction for each new feature. To do so, we transformed each model’s Shapely values by dividing each value by the sum off all Shapely values for the model. The resulting proportional feature contribution for each model were then combined by multiplying each model’s set of Shapely values, by the model’s relative contribution to the overall predictive method.

## Figures and Tables

**Table 1:** Predictive Model Results

Model	AUC ROC Score	Specificity	Sensitivity	PPV
EHR, Genetic, and Survey Data	0.855 +/− 0.060	0.867 +/− 0.130	0.692 +/− 0.021	0.149 +/− 0.082
EHR and Survey Data	0.842 +/− 0.064	0.869 +/− 0.115	0.698 +/− 0.026	0.145 +/− 0.077
EHR and Genetic Data	0.745 +/− 0.052	0.636 +/− 0.122	0.686 +/− 0.011	0.038+/− 0.015
EHR data alone	0.721 +/− 0.053	0.700 +/− 0.078	0.686 +/− 0.011	0.043 +/− 0.010

EHR = electronic health record; AUC = area under the curve; ROC = receiver operating characteristics; PPV = positive predictive value

## Data Availability

The All of Us dataset can be accessed through the Researcher Workbench by following the detailed data application process outlined at https://www.researchallofus.org.

## References

[1] Yang C. & Tebbutt S. J. Long COVID: the next public health crisis is already on its way. The Lancet Regional Health – Europe 28, (2023). 10.1016/j.lanepe.2023.100612 PMC10006728 37131860

[2] WHO Coronavirus (COVID-19) Dashboard. https://covid19.who.int.

[3] Pfaff E. R. Identifying who has long COVID in the USA: a machine learning approach using N3C data. Lancet Digit Health 4, e532–e541 (2022). 35589549 10.1016/S2589-7500(22)00048-6 PMC9110014

[4] Pfaff E. R. De-black-boxing health AI: demonstrating reproducible machine learning computable phenotypes using the N3C-RECOVER Long COVID model in the All of Us data repository. J Am Med Inform Assoc 30, 1305–1312 (2023). 37218289 10.1093/jamia/ocad077 PMC10280348

[5] Lammi V. Genome-wide Association Study of Long COVID. 2023.06.29.23292056 Preprint at 10.1101/2023.06.29.23292056 (2023).

[6] Covid-19 HGI Browser. https://app.covid19hg.org/variants.

[7] Daily Step Counts Before and After the COVID-19 Pandemic Among All of Us Research Participants | Nutrition, Obesity, Exercise | JAMA Network Open | JAMA Network. https://jamanetwork.com/journals/jamanetworkopen/fullarticle/2802674. 10.1001/jamanetworkopen.2023.3526 PMC10028484 36939705

[8] Lukkahatai N., Rodney T., Ling C., Daniel B. & Han H.-R. Long COVID in the context of social determinants of health. Front Public Health 11, 1098443 (2023). 37056649 10.3389/fpubh.2023.1098443 PMC10088562

[9] All of Us Research Program Investigators The ‘All of Us’ Research Program. N Engl J Med 381, 668–676 (2019). 31412182 10.1056/NEJMsr1809937 PMC8291101

[10] Crenshaw K. Demarginalizing the Intersection of Race and Sex: A Black Feminist Critique of Antidiscrimination Doctrine, Feminist Theory and Antiracist Politics.

[11] Crenshaw K. Mapping the Margins: Intersectionality, Identity Politics, and Violence against Women of Color. Stanford Law Review 43, 1241–1299 (1991).

[12] Collins P. Black Feminist Thought | Knowledge, Consciousness, and the Politics of. 10.4324/9780203900055/black-feminist-thought-patricia-hill-collins.

[13] Lett E. & La Cava W. G. Translating intersectionality to fair machine learning in health sciences. Nat Mach Intell 5, 476–479 (2023). 37600144 10.1038/s42256-023-00651-3 PMC10437125

[14] Brannock M. D. Long COVID risk and pre-COVID vaccination in an EHR-based cohort study from the RECOVER program. Nat Commun 14, 2914 (2023). 37217471 10.1038/s41467-023-38388-7 PMC10201472

